# Peroxiredoxins in Colorectal Cancer: Predictive Biomarkers of Radiation Response and Therapeutic Targets to Increase Radiation Sensitivity?

**DOI:** 10.3390/antiox7100136

**Published:** 2018-10-05

**Authors:** Jesse Fischer, Tim W. Eglinton, Frank A. Frizelle, Mark B. Hampton

**Affiliations:** 1 Department of Surgery, University of Otago, Christchurch 8011, New Zealand; tim.eglinton@cdhb.health.nz (T.W.E.); frank.frizelle@cdhb.health.nz (F.A.F.); 2 Centre for Free Radical Research, Department of Pathology and Biomedical Science, University of Otago, Christchurch 8011, New Zealand

**Keywords:** peroxiredoxin, neoadjuvant radiotherapy, colorectal cancer, ionizing radiation, radiation sensitivity, oxidative stress

## Abstract

Colorectal cancer (CRC) is the third most common cancer in the Western world, with one-third of cases located in the rectum. Preoperative radiotherapy is the standard of care for many patients with rectal cancer but has a highly variable response rate. The ability to predict response would be of great clinical utility. The response of cells to ionizing radiation is known to involve immediate damage to biomolecules and more sustained disruption of redox homeostasis leading to cell death. The peroxiredoxins are an important group of thiol-dependent antioxidants involved in protecting cells from oxidative stress and regulating signaling pathways involved in cellular responses to oxidative stress. All six human peroxiredoxins have shown increased expression in CRC and may be associated with clinicopathological features and tumor response to ionizing radiation. Peroxiredoxins can act as markers of oxidative stress in various biological systems but they have not been investigated in this capacity in CRC. As such, there is currently insufficient evidence to support the role of peroxiredoxins as clinical biomarkers, but it is an area worthy of investigation. Future research should focus on the in vivo response of rectal cancer to radiotherapy and the redox status of peroxiredoxins in rectal cancer cells, in order to predict response to radiotherapy. The peroxiredoxin system is also a potential therapeutic target for CRC.

## 1. Introduction

Radiotherapy is a vital tool in cancer therapy and is used in the treatment of a wide range of malignancies including gastrointestinal, genitourinary, head and neck, central nervous system and skin cancer. Radiotherapy may be used as a sole treatment, or in a neoadjuvant or adjuvant setting when combined with surgery. Radiotherapy is used commonly for rectal cancer due to its proven benefit in reducing the rate of local recurrence, but the response is highly variable, with approximately 20% of patients experiencing a pathological complete response (pCR), and up to 40% demonstrating minimal regression or even tumor progression [1]. The ability to predict the response to radiotherapy could crucially inform the decision when considering radiotherapy for rectal cancer. Patients likely to experience a poor response would be best to proceed straight to surgery, thereby avoiding treatment delay and the morbidity of radiotherapy; those predicted to have a good response would be best to receive radiotherapy and may even be considered for non-operative management if a complete clinical response is achieved, thereby avoiding the significant risk of mortality and morbidity with rectal cancer surgery. 

Ionizing radiation (IR) kills cells by direct damage to biomolecules and the generation of reactive oxygen species during the radiolysis of water [2]. This immediate damage is not the only challenge faced by irradiated cells. Redox homeostasis can be disrupted for several weeks, compromising the viability of progeny and bystander cells. While the exact mechanisms of redox disruption are unclear, irreparable damage to nuclear and mitochondrial DNA is thought to increase cellular oxidant production and/or compromise antioxidant defenses, ultimately leading to sustained oxidative stress and cell death [3].

**Figure 1:**
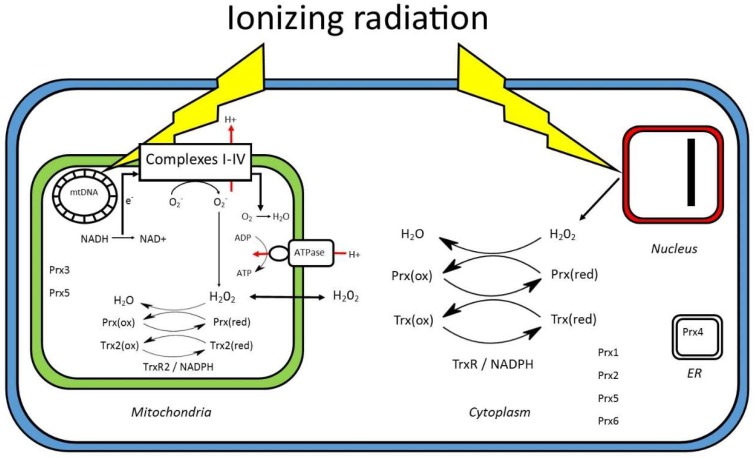
Overview of disrupted redox homeostasis and peroxiredoxin activity following the exposure of cells to ionizing radiation.

Cells irradiated in the absence of oxygen are considerably more resistant to IR [4], confirming the importance of oxidative stress. The increased expression of manganese superoxide dismutase (MnSOD) and mitochondria-targeted catalase have both been shown to protect against IR-induced cell death [5,6,7,8], and chronic glutathione depletion increases radiosensitivity [9]. In this review, we focus on the peroxiredoxin family of antioxidant proteins (Figure 1). These thiol-dependent peroxidases are abundant in mammalian cells and effectively reduce hydroperoxides [10,11]. Humans express six different peroxiredoxins (Prx1-6) with varying cellular locations: peroxiredoxin 1, 2, and 6 are present in the cytoplasm and nucleus; peroxiredoxin 3 present solely in mitochondria; and peroxiredoxin 4 present solely in the endoplasmic reticulum and peroxiredoxin 5 in the cytoplasm, mitochondria, and peroxisomes [12]. The catalytic activity of the peroxiredoxins is dependent on an active cysteine site that is oxidized to a sulfenic acid by hydroperoxides. For Prxs 1–4, a resolving Cys on the second subunit of the homodimer forms an intermolecular disulfide bond. Conversion back to the reduced state requires thioredoxin or glutaredoxin activity, and in cells under increased oxidative stress, the oxidized forms accumulate. In various systems, we have observed that the redox status of endogenous peroxiredoxins can act as a sensitive biomarker of redox homeostasis [13,14]. 

All peroxiredoxins have been shown to have altered expression in human cancer [12]. The aim of this article is to review the role peroxiredoxins play in radiation sensitivity for colorectal cancer (CRC), their potential as predictive biomarkers of radiation sensitivity, and to consider the therapeutic implications. 

## 2. Radiation Therapy for Colorectal Cancer

Approximately one-third of cases of CRC are of rectal origin; with rectal cancer affecting about one in 60 adults in the Western world [15]. While colon and rectal tissue is histologically similar, the clinical behavior and management of colon and rectal cancer differs significantly and they have also demonstrated different outcomes to adjuvant chemotherapy in a clinical setting [16]. Colon cancer is usually treated with bowel resection with or without adjuvant chemotherapy and radiotherapy is rarely used, in contrast with rectal cancer treatment where radiotherapy is common. Historically, local recurrence has been a significant issue following surgery for rectal cancer. The development of improved surgical technique with total mesorectal excision and the use of preoperative radiation therapy has significantly reduced local recurrence rates [17,18]. In addition, the anatomical arrangement of the rectum in the pelvis away from small bowel allows for tumor targeting with less radiation delivery to the vulnerable small bowel. The addition of fluoropyrimidine-based chemotherapy such as 5-fluorouracil to a radiotherapy regimen improves the effectiveness of this treatment [19]. The highly variable response rate of rectal cancer to radiotherapy is however a major challenge in the management of patients with the disease.

There is a significant shift occurring in the approach to rectal cancer treatment. In selected cases, organ preservation (i.e., omitting resection of the rectum) is offered after neoadjuvant chemoradiotherapy if a complete clinical response (determined by clinical and endoscopic examination, and radiological re-assessment with MRI) is achieved. The omission of surgery has major implications in that the chance for early cure with surgery may be missed, and death from rectal cancer progression may result. The benefits of omitting surgery are a reduction in the morbidity and mortality resulting from surgery. For rectal cancer surgery, these risks are significant, with an in-hospital mortality rate of 1–2%, with over 30% of patients having significant post-operative complications and 20–30% of patients requiring a permanent stoma [20]. Similarly, radiotherapy also carries significant morbidity [21,22], and if a poor response could be reliably predicted, then the omission of radiotherapy would be in the patient’s best interests and they should proceed directly to surgical resection. There has been a large volume of research investigating predictors of pCR which can be classified as clinicopathological, radiological and biomarker-related, but no robust predictors have been identified [1].

## 3. Redox Homeostasis, Mitochondria and Radiosensitivity

The irradiation of cells causes direct damage to biomolecules and the radiolysis of water. Within a fraction of a second a series of reactive radical species are generated, and in the presence of oxygen this results primarily in superoxide, hydrogen peroxide and hydroxyl radical formation, and reactive nitrogen species [2]. As well as this early burst, a persistent increase in oxidative stress, for hours to days after radiation exposure, has been reported [2]. This sustained stress, if not lethal, is passed to daughter cells, implicating alterations to nuclear or mitochondrial genomes [3].

Mitochondria are prominent sources of reactive oxygen species, and targets of oxidative stress, and are hypothesized to be a major target of injury by radiation. In 1965, Goldfeder first hypothesized that mitochondria play a role in radiosensitivity, based on the fact that cells with large numbers of mitochondria still function if irradiation compromises a substantial proportion of them [23]. Mitochondrial DNA appears to be more susceptible to damage by IR and chemically-induced oxidative stress [3]. This DNA codes subunits of the electron transport chain (ETC) [24,25,26,27], an important site of superoxide production [28]. Multiple experimental studies have shown that IR directly impacts ETC complexes, disrupting oxidative phosphorylation and ATP synthesis [3]. 

Leach et al. showed that when osteosarcoma cells lacking mitochondrial DNA were irradiated, there was no increase in secondary redox disruption, supporting a central role for mitochondria [29]. Leach et al. also found that the calcium binding protein calbindin limited redox changes, suggesting that calcium played a role in the secondary response [29]. Signaling between mitochondria and the nucleus may also be affected by IR; elevation in a marker of nuclear DNA damage was shown five minutes after nuclear targeting with microscopic irradiation, compared to three hours after cytoplasmic irradiation [29]. Furthermore, the bystander effect was not observed when cells deficient in mitochondrial DNA were used, suggesting mitochondrial function was an essential element of intercellular signaling. Richardson and Harper found that uncoupling the ETC lowered oxidant production and decreased radiosensitivity, especially for hypoxic tumors [30]. They demonstrated that damage to oxygenated tissue is related to mitochondrial oxygen consumption and the production of oxidants, and argued the primary radiation targets in oxygenated tissues are mitochondria that in turn target nuclear DNA.

Cellular antioxidant systems are responsible for maintaining redox homeostasis and protecting against the effects of oxidative stress, including DNA damage [31]. As such, overexpression of endogenous antioxidants can protect cells from radiation-induced injury. This effect was most significant for MnSOD, slight for glutathione peroxidase, while copper-zinc superoxide dismutase (Cu,Zn-SOD) appeared to make no difference [32,33,34]. The fact that MnSOD resides in mitochondria while Cu,Zn-SOD is in the cytosol is consistent with mitochondrial damage playing a key role in radiation-induced injury.

The role of dietary antioxidants in cancer therapy remains unclear [35]. In contrast to cellular enzymes, is it difficult for small molecule oxidant scavengers to reach sufficient concentrations at intracellular sites to have significant impact. Antioxidant supplementation has been reported to reduce side effects from chemotherapy [36], but this could potentially result in decreased treatment efficacy by reducing the oxidative damage that triggers cancer cell death. For example, the DNA-damaging ability of phenolic phytochemicals was shown to be inhibited by ascorbate and N-acetylcysteine in colon cancer cells [37], suggesting that antioxidants can modulate the response to DNA-damaging agents. There is currently no defined role for antioxidant supplementation in colorectal cancer patients.

Differences in radiosensitivity have been found in cells of variable peroxiredoxin expression, and a protective effect against radiation has been found with increased Prx1, Prx2 and Prx4 expression [38]. Peroxiredoxins were proposed as a novel target for radiotherapy by Zhang et al. [38] on the basis of the expression induction by IR in a wide range of cell lines, including human HT29 colon cancer cells, as well as tissue including colorectal and non-colorectal tumors [39,40,41,42,43,44] and an association between expression status and radiosensitivity of tumor cells including decreased radiosensitivity after the knockdown of peroxiredoxins [44,45].

## 4. Peroxiredoxins and Colorectal Cancer

There have been several studies examining peroxiredoxin expression in CRC (Table 1), with the expression of all six peroxiredoxins reported to be increased [46]. We review these studies, with particular focus on radiosensitivity for each peroxiredoxin. 

### 4.1. Peroxiredoxin 1

Prx1 expression is increased in CRC and has been suggested as a prognostic and predictive biomarker for rectal cancer on the basis of both in vitro and in vivo studies conducted by Chen et al. [47]. Prx1 expression as evaluated by immunohistochemistry (IHC) was significantly associated with a poor pathological response rate for 120 human subjects with rectal cancer treated with radiotherapy, with a response rate of 43.6% when there was negative staining and 20% when there was positive staining; this effect was accentuated when p53 staining was negative. Prx1 suppression by a Prx1 silencing vector increased radiosensitivity of HT-29 and HCT-116 colon cancer cell lines and inhibited tumor growth in a mouse model. Prx1 expression was also a significant predictor of disease free survival (DFS) in the group of patients who were p53 negative. The authors’ conclusion that Prx1 expression was associated with both poorer response to treatment and poorer prognosis appears justified but little work has been done to further investigate this. 

### 4.2. Peroxiredoxin 2

Peng et al demonstrated that both Prx2 mRNA and protein content was higher in CRC cell lines than normal colonic epithelial cells, and Prx2 expression was significantly upregulated in human CRC tissue compared with adjacent non-cancerous tissue [48]. They also assessed clinicopathological correlation and identified an association between increased Prx2 expression and poor histological differentiation, advanced local invasion, lymph node metastases and advanced tumor node metastasis stage, as well as shorter DFS, suggesting it may have a useful role as a prognostic marker for CRC [48]. In support of Prx2 expression as a stimulator of cancer progression, when a tumor knockdown of Prx2 was performed in a mouse model it was found to inhibit CRC cell growth, and when Prx2 silencing was performed in both a polyposis mouse model and human CRC cell lines, mouse polyposis was decreased by a reduction in beta-catenin as an end-point, and beta-catenin levels are reduced in the cells in which Prx2 is silenced [49]. This suggests a mechanism of action involving the canonical Wnt signaling pathway. Although this is one of many molecular pathways in CRC development, the canonical Wnt pathway is disrupted in familial adenomatous polyposis due to germline mutations of the adenomatous polyposis coli gene, and commonly disrupted in sporadic CRC development. This raises the exciting possibility of therapeutic agents to limit polyposis progression in patients with familial adenomatous polyposis, as well as to modify the risk of sporadic CRC. Lu et al. also found that Prx2 was upregulated in CRC and contributed to CRC cell survival by protecting cells from oxidative stress [51].

In contrast to the above findings, an earlier study by Ji et al. examined the mRNA and protein expression of Prx2 in CRC tissue of 137 patients and found lower Prx2 expression was associated with poor differentiation, advanced cancer stage and poorer survival. They also looked for a correlation between serum Prx2 and OS or DFS and found none [52]. There is no clear explanation of the difference between this study and that of Peng et al.

The silencing of Prx2 expression has been shown to sensitize colon cancer cell lines to 5-fluoruracil by facilitating cell death and apoptosis [53], and also to sensitize colon cancer cells to IR [44]. Despite promising work with cell lines and xenograft models, there has been no investigation of Prx2 as a biomarker for pathological response to radiotherapy for rectal cancer in human subjects in vivo.

### 4.3. Peroxiredoxin 3

Prx3 is the only mammalian peroxiredoxin that is present exclusively in mitochondria [58]. Song et al. collected tumor tissue from eight patients with colon cancer and investigated Prx3 expression using immunofluorescent and quantitative techniques [54]. They found increased expression in colon cancer stem cells compared with normal colorectal tissue stem cells, and that cell death was not increased with escalating 5-fluoruracil dosing in colon cancer stem cells, showing some resistance to chemotherapeutic action. This suggests a cell survival advantage associated with Prx3 expression. The effect of Prx3 expression on radiosensitivity in CRC has not been examined, despite the importance of mitochondria in the response to IR.

### 4.4. Peroxiredoxin 4

The endoplasmic reticulum protein Prx4 has also been linked to CRC. Prx4 expression was higher in CRC tissue than normal colorectal tissue assessed with IHC and qPCR techniques, and increased Prx4 expression also correlated with negative clinical factors including depth of invasion and stage [55]. In contrast, a small study that looked at peroxiredoxins in eight patients with CRC found Prx4 trending towards a lower positivity rate in CRC tumor tissue than normal controls and had no association with clinical stage or lymph node metastases [46]. Prx4 expression by western blotting was slightly higher in normal control tissue than CRC tissue. Both these studies included small numbers of samples and statistical significance for positivity was not achieved in the second study, so this inconsistency may be a reflection of inadequate sample size.

An exploratory study investigating novel markers predicting pathological response to chemoradiotherapy for rectal cancer using a 2D-DIGE (difference gel electrophoresis) quantitative proteomic approach in 35 patients with rectal cancer found higher Prx4 expression in pre-treatment tumor samples in poor responders to chemoradiotherapy, suggesting a potential role as a predictive biomarker of response to chemoradiotherapy for rectal cancer [56]. There were no differences seen in any of the other peroxiredoxins in this study.

### 4.5. Peroxiredoxin 5

There is limited literature available on the role of Prx5 in CRC, but a recent study demonstrated increased expression of Prx5 in colon cancer cell lines is associated with cell proliferation, migration and invasion, while decreased expression had the inverse effect [57]. This study also found enhanced tumor growth with increased expression of Prx5 in a xenograft mouse model. There are no reports relating to Prx5 in human CRC.

### 4.6. Peroxiredoxin 6

Wu et al. demonstrated Prx6 expression positivity in 56% of CRC tissue vs 12.5% of normal control tissue and a significantly higher expression in CRC tissue with western blotting, but no association with clinical stage or lymph node metastases [46]. There are no other reports linking Prx6 to CRC development, progression or treatment response. 

## 5. Peroxiredoxins as Prognostic and Predictive Biomarkers

Tumor biology is often the most important determinant of patient outcome, and tumor features can be useful for predicting the natural history of CRC. There is significant evidence to support an association between increased Prx2 expression and poor prognostic factors such as more advanced tumor stage and decreased survival. Prx1 has also been associated with clinical outcomes. Peroxiredoxins may be one marker of the underlying tumor biology as redox homeostasis itself is critical to cell survival.

The studies described above suggest that peroxiredoxin expression level may have a role in predicting radiosensitivity and/or chemosensitivity for CRC. The majority of this evidence is based on work with cell lines or animal models, with limited evidence of an in vivo response to treatment in human subjects. Only Prx1 and Prx4 have been linked to radiotherapy response for rectal cancer in vivo, and each by a single study. Prx2 has not yet been linked to response to radiotherapy for rectal cancer but an apparent role in the development, progression and in vitro response to chemo/radiotherapy of CRC makes it a good candidate for further investigation.

Peroxiredoxins are sensitive markers of cellular redox homeostasis. Typical 2-Cys peroxiredoxins can be present in oxidized homodimers (~40kD) or reduced monomers (~20kD), with the oxidized form accumulating in cells due to either increased rates of hydroperoxide generation or limitations in the rate of reduction of the oxidized forms [14]. A simple method of measuring the oxidized and reduced forms of peroxiredoxins exists by western blotting of samples in which proteins have been separated by non-reducing polyacrylamide gel electrophoresis, and the relative ratio of oxidized and reduced peroxiredoxin calculated. This methodology has been shown to be valuable in measuring oxidative stress in erythrocytes and cardiac tissue [14] and in cultured cells treated with cytotoxic agents such as auranofin and phenethyl isothiocyanate [59,60]. In the studies to date investigating peroxiredoxins in CRC, total peroxiredoxin expression has been assessed, but not the redox status. Indeed, no comprehensive analysis of peroxiredoxin redox status in tumor material has been reported. 

## 6. Peroxiredoxins as Therapeutic Targets

Work on expression silencing in laboratory models suggests peroxiredoxin inhibition as a possible therapeutic strategy. Various peroxiredoxin inhibitors have been described [61,62,63], including inhibitors of mitochondrial Prx3 [64], but there have been no studies specifically addressing this in CRC. The inhibition of peroxiredoxins in CRC could result in increased oxidative stress during IR and may even have direct anti-tumor activity. Another potential therapeutic target is the thioredoxin system, which is important in maintaining peroxiredoxins in their reduced form. Thioredoxin reductase inhibitors such as auranofin have been shown to result in cell death due to mitochondrial dysfunction and hydrogen peroxide accumulation in the context of neurological disorders [65] and have been proposed as anti-cancer agents based on upregulation in advanced malignancy and impairment of tumor growth in human tumor xenografts in mouse models [66]. A thioredoxin-1 inhibitor has been shown to inhibit the growth and progression of CRC cell lines [67], and while there has been no examination of radiosensitivity, this would be worthy of investigation. 

## 7. Summary

Predicting the radiation sensitivity of rectal cancer carries enormous clinical significance, particularly in the setting of an evolving organ-preservation approach to management. Tools to assist in management decisions regarding organ-preservation strategies would be of important clinical value. Mitochondrial function including redox homeostasis is integral to the cellular response to IR, and peroxiredoxins are important players in these systems.

There is evidence of increased expression of all six peroxiredoxins in CRC, albeit with some inconsistencies among reported associations for Prx2 and Prx4. These inconsistencies indicate a need for further research. Prx1, Prx2 and Prx4 appear the most promising as prognostic indicators and/or predictive biomarkers of response to radiotherapy for CRC based on the available evidence. Prx3, Prx5 and Prx6 have limited data to support roles as markers, but what is available does suggest increased expression in CRC, and the role of these enzymes in CRC is in need of further investigation. Prx3 is of particular interest as the only peroxiredoxin present exclusively in mitochondria, given the central role of mitochondria in the response to IR.

The potential to increase cancer cell death and the chance of pathological complete response from radiotherapy for rectal cancer is real. Neoadjuvant radiotherapy is commonly given with fluoropyrimidine-based therapy as a radiosensitizer. It is possible that the modulation of the peroxiredoxin/thioredoxin system could improve response to chemoradiotherapy for rectal cancer by acting on both radiotherapeutic and chemotherapeutic pathways. 

It is difficult to compare the radiotherapy response for colon and rectal tumors due to the different uses of radiotherapy in the two sites, and because the clinical behavior of colon and rectal cancer differs significantly despite their histological similarity. The clinical utility of radiotherapy is much greater for rectal cancer than colon cancer; therefore, further investigation of the radiotherapy response associated with peroxiredoxin expression and redox status would be best studied in human subjects with rectal cancer if the potential for clinical translation is to be maximized. 

## Figures and Tables

**Table 1:** Peroxiredoxin expression and associations with radiosensitivity and prognosis of colorectal cancer.

Gene Name	Expression in CRC	Radiosensitivity	Prognostic Indicator	Predictor of Pathological Tumor Response	Reference(s)
PRDX1	↑	↑ expression → ↓ radiosensitivity	Yes	↑ expression → ↓ response	[47]
PRDX2	↑	↓ expression → ↑ radiosensitivity	Yes	-	[48,49,50,51,52,53]
PRDX3	↑	-	-	-	[54]
PRDX4	↑ / ↓	-	Yes	Yes	[46,55,56]
PRDX5	↑	-	-	-	[57]
PRDX6	↑	-	-	-	[46]

Legend: ↑ = increased, ↓ = decreased, → = association between expression and radiosensitivity or tumor response. CRC: colorectal cancer. PRDX: peroxiredoxin gene.
